# Regular sports services: Dataset of demographic, frequency and service level agreement

**DOI:** 10.1016/j.dib.2021.107054

**Published:** 2021-04-20

**Authors:** Paulo Pinheiro, Luís Cavique

**Affiliations:** a CEDIS, Lisboa, Portugal; b Universidade Aberta, Lisboa, Portugal/Universidade de Trás-os-Montes e Alto Douro, Vila Real, Portugal; c LASIGE, FCUL, Lisboa, Portugal

**Keywords:** Sport services, Customer retention, Machine learning, Actionable knowledge

## Abstract

This article describes a dataset of different services acquired by users during the period in which they are active in a sports facility as well as their behavior in terms of frequency of the sport facility itself and the type of classes they prefer to attend. Each observation in the dataset corresponds to one user, including the features of subscriptions and frequency.

Data were collected between June 1st 2014 and October 31st 2019 from a database of an ERP solution operating in a sports facility in Lisbon, Portugal. From this database, it was possible to perform operations of extraction, transformation and loading into the dataset.

The dataset with real data can be useful for research in areas such as customer retention, machine learning, marketing, actionable knowledge and others.

Although we present real data from users of a sports facility, in order to comply the GDPR legislation, the attributes that could identify the users were removed making the data anonymized.

## Specifications Table

**Table:** 

Subject	Information Systems and Management
Specific subject area	Regular sport services, Retention, Machine learning
Type of data	Table (csv file)
How data were acquired	Data were acquired from a sport facilities management ERP e@sport system with a Microsoft® SQL*Server database
Data format	Mixed (raw and pre-processed)
Parameters for data collection	This dataset corresponds to actual data from the functioning of a sports facility and refers to all new users who signed up between June 1st 2014 and October 31st 2019.
Description of data collection	Demographic and service level agreement (SLA) data is collected by operators in the process of enrolling users in the activities they intend to practice.
	The data regarding the frequency of the sports facility and classes were obtained by the access control system where each user identifies himself with an RFID card to access the facilities on the days and times agreed in his SLA.
Data source location	A Sport facility in Lisbon, Portugal
Data accessibility	Repository name: Mendeley Data
	Data identification number: https://doi.org/10.17632/yprk4jdgnv.1
	Direct URL to data: https://data.mendeley.com/datasets/yprk4jdgnv/1
Related research article	P. Pinheiro, L. Cavique, An Actionable Knowledge Discovery System in Regular Sports Services, Á. Rocha et al. (Eds.): WorldCIST'19 2019, AISC 931, pp. 461–471, 2019.
	https://doi.org/10.1007/978-3-030-16184-2_44

## Value of the Data

• The data in this paper describe real data of users' involvement with the sports facility they attend at three levels: at the demographic level; in terms of the frequency of installations; and at the level of the service level agreement [1].
• These data can be used to perform research on regular sports services in different problems related to customer retention, customer segmentation, lifetime value, actionable knowledge, causality, among others;
• Machine learning researchers can use the datasets for benchmarking the performance of different algorithms for solving the same type of problem (surviving analysis, dropout classification, customer segmentation, or other); Marketeers can use this data, with or without machine learning help, to trace profiles of sports facilities users;

## 1 Data Description

Nowadays, sports facilities have ERP and access control systems that allow to obtain very rich and real information about the way their users behave with respect to Loyalty and Retention. This dataset contains sets of demographic attributes, attributes related to the contracted services and which can be related to the referred loyalty and retention aspects.

The following paragraphs describe some of the attributes, in order to better understand in which conditions the users are considered dropouts.

In this data set, whose attributes are shown in Table 1, the user is considered active (attribute *Dropout = “False”*) from the first time he signed up (attribute *EnrollmentStart*) until he expressed his willingness to give up or until the moment when, due to lack of payment, he was considered a dropout according to the installation's regulations, in this case two months in debt (attribute *EnrollmentFinish*). Note that as the data set was considered until October 31, 2019, there are users who have the attribute *EnrollmentFinish = “October 31, 2019”*, but who at that date were not dropouts.

**Table 1:** Attributes description.

#	Attribute name	Type	Description
1	Id	Uid	Unique identifier of the record/example

2	Age	Int	Age of the user at October 31st 2019 if it is not a dropout, or age of the user at date specified in attribute *EnrollmentFinish* if it is a dropout

3	AgeClass2	Categorical	Age classified in age groups. The attribute can have the following values: “[00,20[“ for users under 20 years old; “[20,35[” for users aged between 20 and 34; “[35,49[” for users aged between 35 and 48 years; “[49,65[” for users aged between 49 and 64 years; “[65,inf[” for users aged 65 and over;

4	Gender	Categorical	Gender of the user (*Male* or *Female*)

5	NumberOfReferences	Int	Number of people with which the user is related by family relationship or friendship

6	HasReferences	Boolean	This field contains the value *True* if *NumberOfReferences> 0*, or *False* otherwise

7	EnrollmentStart	Date	Date of first enrollment

8	EnrollmentFinish	Date	Finish date of last enrollment

9	EnrollmentDuration	Int	Difference, in months, between start of first enrollment (*EnrollmentStart*) and finish date of last enrollment (*EnrollmentFinish*)

10	EnrollmentDurationClass1	Categorical	Contains the class obtained with the Hughes method for the values of the attribute *EnrollmentDuration*

11	EnrollmentDurationClass2	Categorical	Contains the class obtained for the values of attribute *EnrollmentDuration* according to the following ranges: “[00,01]” for between 0 and 1 month; “]01,02]” for less then 2 months; “]02,04]” for between 3 and 4 months; “]04,06]” for between 5 and 6 months; “]06,09]” for between 7 and 9 months; “]09,12]” for between 10 and 12 months; “]12,inf[” for more than 12 months;

12	DateLastVisit	Date	Date and time of the user's last visit to the sport facility

13	DaysWithoutFrequency	Int	Number of days the user did not visit the facilty before being considered a dropout

14	DaysWithoutFrequencyClass1	Categorical	Contains the class obtained with the Hughes method for the values of the attribute *DaysWithoutFrequency*

15	DaysWithoutFrequencyClass2	Categorical	Contains the class obtained for the values of attribute *DaysWithoutFrequency* according to the following ranges: “[00,07]” for between 0 and 7 days (1 week) without attending;
			“]07,15]” for 8 to 15 days (2 weeks) without attending; “]15,30]” for between 16 and 30 days (approx. between 16 days and 1 month) without attending; “]30,60]” for between 31 and 60 days (approx. between 1 and 2 months) without attending; “]60,inf]” for more than 60 days without attending;

16	LifetimeValue	Numeric	Total amount paid by the customer during the period in which he was enrolled (between *EnrollmentStart* and *EnrollmentFinish*)

17	LifetimeValueClass1	Categorical	Contains the class obtained with the Hughes method for the values of the attribute *LifetimeValue*

18	AverageFrequency	Numeric	Average weekly frequency throughout the period in which the user was enrolled (between *EnrollmentStart* and *EnrollmentFinish*)

19	AverageFrequencyClass1	Categorical	Contains the class obtained with the Hughes method for the values of the attribute *AverageFrequency*

20	AverageFrequencyClass2	Categorical	Contains the class obtained for the values of attribute *AverageFrequency* according to the following ranges: “[0.0,0.1]” for less than or equal to 0.1 times a week; “]0.1,0.2]” for less than or equal to 0.2 times a week; “]0.2,0.5]” for less than or equal to 0.5 times a week; “]0.5,1.0]” for less than or equal to 1 time a week; “]1.0,2.0]” for less than or equal to 2 times a week; “]2.0,3.0]” for less than or equal to 3 times a week; “]3.0,inf[” for more than 3 times a week;

21	UseByTime	Boolean	Indicates whether the user was enrolled in this form of use (*True* if he was, *False* otherwise)

22	AthleticsActivities	Boolean	Indicates if the user was ever enrolled in athletics during the period between *EnrollmentStart* and *EnrollmentFinish* (*True* if it was, *False* otherwise)

23	WaterActivities	Boolean	Indicates if the user was ever enrolled in athletics during the period between *EnrollmentStart* and *EnrollmentFinish* (*True* if it was, *False* otherwise)

24	FitnessActivities	Boolean	Indicates if the user was ever enrolled in swimming during the period between *EnrollmentStart* and *EnrollmentFinish* (*True* if it was, *False* otherwise)

25	TeamActivities	Boolean	Indicates if the user was ever enrolled in swimming during the period between *EnrollmentStart* and *EnrollmentFinish* (*True* if it was, *False* otherwise)

26	RacketActivities	Boolean	Indicates whether the user was enrolled in team sports during the period between *EnrollmentStart* and *EnrollmentFinish* (*True* if it was, *False* otherwise)

27	CombatActivities	Boolean	Indicates if the user was enrolled in combat sports during the period between *EnrollmentStart* and *EnrollmentFinish* (*True* if it was, *False* otherwise)

28	SpecialActivities	Boolean	Indicates if the user was enrolled in sports for disabled people during the period between *EnrollmentStart* and *EnrollmentFinish* (*True* if it was, *False* otherwise)

29	OtherActivities	Boolean	Indicates if the user was enrolled in any activity that does not fit into any of the other previous activities mentioned in attributes #22 to 28 during the period between *EnrollmentStart* and *EnrollmentFinish* (*True* if it was, *False* otherwise)

30	NumberOfActivities	Int	It represents the number of activities in which the user was enrolled during the period between *EnrollmentStart* and *EnrollmentFinish*. In practice, it corresponds to the counting of attributes from #22 to 29 with *True* value

31	NumberOfFrequencies	Int	Number of visits to the sports facility since the date indicated in *EnrollmentStart* and the date indicated in *EnrollmentFinish*

32	NumberOfFrequenciesClass1	Categorical	Contains the class obtained with the Hughes method for the values of the attribute *NumberOfFrequencies*

33	AttendedClasses	Int	Number of classes the user attended between *EnrollmentStart* and *EnrollmentFinish*

34	AttendedClassesClass1	Categorical	Contains the class obtained with the Hughes method for the values of the attribute *AttendedClasses*

35	AttendedClassesAverage	Numeric	Represents the average classes attended per week

36	AttendedClassesAverageClass1	Categorical	Contains the class obtained with the Hughes method for the values of the attribute *AttendedClassesAverage*

37	NumberOfRenewals	Int	Number of renewals during the registration period (between *EnrollmentStart* and *EnrollmentFinish*)

38	LastPeriodStart	Date	Start date of the last activity or the last two months if less

39	LastPeriodFinish	Date	End date of last activity or last two months if less

40	AllowedWeeklyVisitsBySLA	Int	Indicates the number of weekly visits that the user can make to the facilities according to the service he had hired in the last 2 months of his registration (between *LastPeriodStart* and *LastPeriodFinish*)

41	AllowedNumberOfVisitsBySLA	Int	Indicates the total number of visits that the user can make to the facilities according to the service he had hired in the last 2 months of his registration (between *LastPeriodStart* and *LastPeriodFinish*)

42	RealNumberOfVisits	Int	Indicates the actual number of visits that the user made to the facilities in the last two months of his registration (between *LastPeriodStart* and *LastPeriodFinish*)

43	Ratio	Numeric	Indicates the relationship between the actual number of visits the user has made and the number of visits the user could have made. The following formula is used (number represent attribute id): 43 = (42 / 41) * 100

44	RatioClass2	Categorical	Contains the class obtained for the values of attribute *Ratio* according to the following ranges: “[0]” “]0,25]” for 25% or less; “]25,50]” for 50% or less; “]50,75]” for 75% or less; “]75,100]” for more than 75%;

45	Dropout	Boolean	Represents the user's status on the date indicated in *EnrollmentFinish*, assuming the value *True* if he is quitting or *False* if he is not

**Table 2:** Date attributes.

Attribute	Min	Median	Max
EnrollmentStart	2014/06/02	2016/09/23	2019/10/21
EnrollmentFinish	2014/07/11	2018/06/04	2019/10/31
LastPeriodStart	2014/06/02	2018/02/07	2019/10/30
LastPeriodFinish	2014/07/11	2018/04/07	2019/10/31
DateLastVisit	2014/07/03	2018/01/18	2019/10/31

This sports facility closes one month in the summer (August) due to the need to maintain its equipment, namely the swimming pools. Because of this closure, users subscribe to regular sports services for the period between September of one year and July of the following year in a similar way to school periods. If the user wishes to continue to attend the installation in the following season, he must renew it. The *NumberOfRenewals* attribute indicates how many times the user has renewed their registration between the period defined by *EnrollmentStart* and *EnrollmentFinish*.

There are activities whose forms of adherence allow users to attend every day of the week at the hours that the user wishes, the so-called free transit, and other activities in which the user has to choose the days of the week and times (the classes) that intends to attend.

**Fig. 1:**
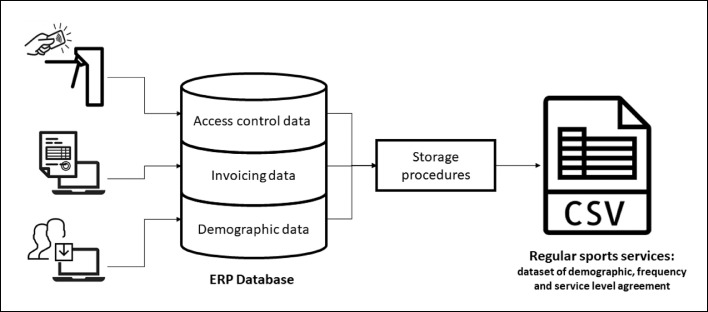
Architecture of the data collection system and dataset construction process.

**Fig. 2:**
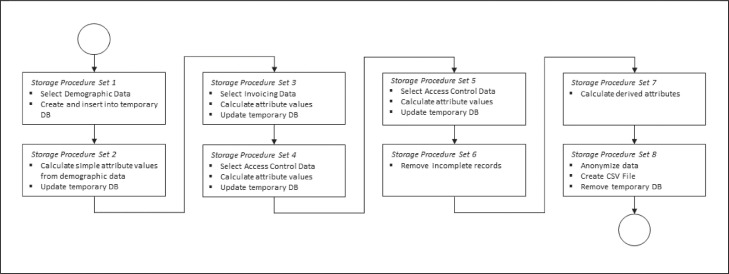
Pipeline of storage procedures in Microsoft® SQL*Server.

Users who are registered in free transit have the attribute *AllowedWeeklyVisitsBySLA = 7*, corresponding to the possibility of being able to attend 7 days a week. Users who choose the day(s) and hours of frequency have the attribute *AllowedWeeklyVisitsBySLA = 〈number of days they chose to attend〉*.

There is also a third form of valid use for swimming pools, the so-called free use, in which the user loads his card with a certain amount, and as he uses it, the amount corresponding to the time spent in the facilities is discounted. Users who have had this type of use have the attribute *UseByTime = True*.

**Table 3:** Integer and numeric attributes.

Attribute	Min	1st Q.	Median	3rd Q.	Max	
Age	0	19	23	31	87	
EnrollmentDuration	0	4	9	19	64	
DaysWithoutFrequency	0	13	41	84	1745	
LifetimeValue	0	83.6	166.2	355.1	6727.8	
AverageFrequency	0	0.29	0.57	1.01	4.94	
NumberOfActivities	1	1	1	1	5	
NumberOfFrequencies	1	7	18	46	1031	
AttendedClassesAverage	0	0	0	0.1	3.6	
AllowedWeeklyVisitsBySLA	1	4	7	7	7	
AllowedNumberOfVisitsBySLA	0.56	25.72	38.99	60.97	240.03	
RealNumberOfVisits	0.00	1.00	4.00	7.00	84.00	
Ratio	0.00	3.22	9.99	22.96	100.00	
NumberOfRenewals	0	0	1	2	6	
NumberOfReferences	0	0	0	0	3

**Table 4:** Categorical attributes.

Attribute	Categories	Min	Max	N° of examples
EnrollmentDurationClass1	1 2 3 4 5	0 3 7 11 23	3 7 11 23 64	2989 2989 2988 2988 2988

EnrollmentDurationClass2	[00,01] [01,02] [02,04] [04,06] [06,09] [09,12] [12,inf[	0 2 3 5 7 10 13	1 2 4 6 9 12 64	1065 1138 2135 1588 1788 2012 5216

DaysWithoutFrequencyClass1	1 2 3 4 5	0 9 32 51 106	9 32 50 106 1745	2989 2989 2988 2988 2988

DaysWithoutFrequencyClass2	[00,07] [07,15] [15,30] [30,60] [60,inf[	0 8 16 31 61	7 15 30 60 1745	2745 1455 1639 4214 4889

LifetimeValueClass1	1 2 3 4 5	0.00€ 72.60€ 122.60€ 223.60€ 435.20€	72.60€ 122.60€ 223.60€ 435.20€ 6727.80€	2989 2989 2988 2988 2988

AverageFrequencyClass1	1 2 3 4 5	0.00 0.24 0.45 0.71 1.16	0.24 0.45 0.71 1.15 4.94	2989 2989 2988 2988 2988

AverageFrequencyClass2	[0.0,0.1] [0.1,0.2] [0.2,0.5] [0.5,1.0] [1.0,2.0] [2.0,3.0] [3.0,inf[	0.00 0.11 0.21 0.51 1.01 2.01 3.01	0.10 0.20 0.50 1.00 2.00 3.00 4.94	1088 1459 4131 4507 2891 668 198

NumberOfFrequenciesClass1	1 2 3 4 5	1 5 13 25 57	5 13 25 57 1031	2989 2989 2988 2988 2988

AttendedClassesClass1	1 2 3 4 5	0 0 0 0 9	0 0 0 9 581	2989 2989 2988 2988 2988

AttendedClassesAverageClass1	1 2 3 4 5	0.00 0.00 0.00 0.00 0.24	0.00 0.00 0.00 0.24 3.60	2989 2989 2988 2988 2988

RatioClass2	[0] [0,25] [25,50] [50,75] [75,100]	0.00 0.42 25.00 50.01 75.01	0.00 25.00 50.00 75.00 100.00	2698 9043 2429 568 204

**Table 5:** Boolean attributes.

Attribute	True	False
UseByTime	704	14,238
AthleticsActivities	110	14,832
WaterActivities	4432	10,510
FitnessActivities	8610	6332
TeamActivities	831	14,111
RacketActivities	351	14,591
CombatActivities	1613	13,329
SpecialActivities	397	14,545
OtherActivities	28	14,914
HasReferences	296	14,646
**Dropout**	**11,968**	**2974**

There are several also other aspects to be highlighted about these data:

a) Each record / row in the dataset contains data relating to a single user and there are no duplicate users;
b) Each row has a set of attributes that relate to the entire period in which the user was enrolled, defined by the attributes *EnrollmentStart* and *EnrollmentFinish*, and another set of attributes (attributes from ID 40 to 44) that relate to the last activity in which the user was enrolled or to the last two months of enrollment if less. This period is defined by the attributes *LastPeriodStart* and *LastPeriodFinish*;
c) The attributes whose name ends with “Activities” indicate the type of activities that the user attended during his registration;
d) The attributes whose name ends with “Class1” correspond to the Hughes method classification [2] of the attribute with the corresponding name. Table 4 shows the possible classes, the minimum and maximum values, and the number of examples in each class for each of these attributes;
e) The attributes whose name ends with “Class2” are filled with their own categorization, according to the specialized literature. Possible values are indicated in the “Description” column of the attribute in Table 1. Table 4 shows the possible classes, the minimum and maximum values, and the number of examples in each class for each of these attributes;

Table 1 shows all the attributes of the dataset. The first column indicates the ID of the attribute, the second column indicates the name of the attribute, the third column indicates the type of the attribute and the last column describes the meaning of the values that each attribute can contain.

## 2 Experimental Design, Materials and Methods

The dataset referred to in this article results from a set of extraction, processing and loading operations to the data contained in a Microsoft® SQL*Server database to support a Sports Facilities Management application of a Sports Complex.

This database contains several tables where users' demographic data are recorded, all billing carried out, as well as records of access to the installation through the access control mechanisms available to the installation.

The observation of the diagrams that the original database exposes allowed to verify some guarantees of consistency and integrity of the data. However, since the data is spread over several tables, it was considered useful to synthesize the data in a single table that the present data set reflects, which made the approach made in previous studies simpler, namely in [3]. To carry out this operation, storage procedures were developed that import and summarize the information of each user in a single row and produce de csv file that contains the dataset. These operations were carried out with three main objectives:

a) to synthesize the relevant attributes in just one set of data, in order to simplify the use of the data;
b) to remove records with missing values or inconsistent data;
c) to create new attributes through symbolic-numeric conversions, numeric-symbolic conversions, discretization and others [4] as described in the “Data description” section.

Fig. 1 illustrates the architecture of the data collection system and the dataset construction process.

Fig. 2 illustrates the pipeline of storages procedures executed in Microsoft® SQL*Server from the selection of the initial data to the final step of creating the CSV.

After the execution of all storage procedures, Microsoft® SQL*Server Management Studio Ver. 17.9.1 and RStudio Ver. 1.3.959 were used in order to analyze the resulting data. Records that had missing, inconsistent and/or outlier values were removed from the data set.

In the end, it resulted in a data set with 14,942 records, of which the statistically significant values of the attributes [5] according to their type are shown in the following tables. Thus, Table 2 presents the date attributes, Table 3 the integer and numeric attributes, Table 4 the categorical attributes and Table 5 the Boolean attributes.

## Ethics Statement

The data were collected from a database of a sports center of a higher education institution called Estádio Universitário de Lisboa, Universidade de Lisboa;

The data is anonymized and there is no way to identify to whom they correspond to from the ID attribute of the data set, as it was generated randomly.

The use of anonymized data was permitted by the Lisbon University Stadium of the University of Lisbon within the scope of the publication of this article and under the conditions requested by the Editor.

As a higher education institution, the institution in question is still interested in the knowledge that it may see extracted from these data.

## CRediT Author Statements

**Paulo Pinheiro:** Conceptualization, Methodology, Software, Data curtion, Writing original draft, Visualization; **Luís Cavique:** Supervision, Writing review & editing.

## Declaration of Competing Interest

The authors declare that they have no known competing financial interests or personal relationships which have, or could be perceived to have, influenced the work reported in this article.

## Appendix Supplementary materials

Image, application 1
