# Supplementary material for: Regular sports services: Dataset of demographic, frequency and service level agreement
Source: Data Brief. 2021 Apr 20;36:107054. doi: 10.1016/j.dib.2021.107054 (PMC8100056; doi:10.1016/j.dib.2021.107054)
Supplement: Supplementary file 1 [file mmc1.pdf]

An actionable knowledge discovery system in regular sports services

Paulo Pinheiro [1[0000-0002-8912-2244]] and Luís Cavique [2[0000-0002-5590-1493]]

[1] Master degree student at Universidade Aberta, Cedis
ppinheiro@cedis.pt
[2] Universidade Aberta, BioISI-MAS
luis.cavique@uab.pt

Abstract. This work presents an actionable knowledge discovery system for real user needs with three steps. In the first step, it extracts and transforms existing data in the databases of the ERP and CRM systems of the sports facilities and loads them into a Data Warehouse. In a second phase, predictive models are applied to identify profiles more susceptible to abandonment. Finally, in the third phase, based on the previous models, experimental planning is carried out, with test and control groups, in order to find concrete actions for customer retention.

Keywords: actionable knowledge discovery system; sport services; predictive analysis; experimental planning; loyalty.

1 Introduction

The sports services sector is characterized by a high dropout rate [1]. In Portugal, according to the Fitness Barometer of the Association of Portuguese Gymnasiums and Academies [2] overall dropout rate in 2016 was 69% corresponding to a retention rate of 31%.

Actionable Knowledge is the knowledge required to initiate changes in the operational environment in order to create value. In this customer retention work we reuse some concepts of Database Marketing [3] and IDIC model (Identify, Differentiate, Interact, Customize) [4]. We intend to use existing data to identify the regular sports users services at risk of moving out, differentiating them from the rest through machine learning techniques and to interact with them using personalized loyalty actions. The proposed model, which includes the data preparation, the profile discovery using predictive models and the loyalty actions with evaluation, can be presented in the following schematic form:

$$\text{Data} \rightarrow \text{Models} \rightarrow \text{Loyalty}$$

This document has the following structure. In Section 2, a brief approach is given to a related work that has been done in the area of sports service retention and in the application of predictive analysis in retention. In Section 3, we present the data preparation methodology. In Section 4, we introduce the predictive model applied to the

data presented in the previous section and measure the results obtained. In Section 5, we approach the planning of experiences and the actions of loyalty that can be introduced. In Section 6 we present not only a proposal that allows us to measure the results obtained, but also a conclusion on the effectiveness of the loyalty actions. Finally, in Section 7 we present the contributions of the work.

2 Related work

Studies ([1],[5]) carried out in the area of sports service retention generally conclude that user retention and loyalty is related to the quality of the facilities, staff and the overall quality of services provided, results which have been used by sports facilities management. This type of work is always based on surveys carried out on a sample of the population, which suggests the search for other methods to measure or ensure a lesser character generic and that allows to activate mechanisms directed to users in pre-dropout phase.

In addition to two studies ([6] [7]) applied to fitness and regular sport services, there were no other studies in the area of data mining applied directly to data obtained in sports services. However, the problem of high drop-out rates / low retention rates in other types of services has led to such churn prediction, especially in telecommunications, where high dropout rates are also observed. However, given the large size of databases and costs involved, most studies in this area use small samples of customer records, which may result in poor reliability and validity of the results obtained [8].

In order to focus their efforts on the clients they are most likely to meet and/or those who will be most profitable, companies seek to identify patterns and needs in customer groups (segmentation process). There are simple methods that do not require predictive models are RFM and RM [9] based on properties such as the Recency (when the customer's last visit occurred), Purchasing Frequency and the overall Monetary value.

As segmentation methods that use predictive models Siegel [10] refers to the Lift and Uplift models. Lift identifies customers who are most susceptible to a particular communication or marketing action. Uplift, in order to learn how to distinguish influential clients - those who make a difference in doing some treatment - learns from customers who have been contacted and those who have not been contacted, so it is necessary to use two data sets to train the model, a group of clients who are "treated" - treatment group - and another group of clients who are not - control group. The Uplift method also uses data mining techniques to segment through Uplift trees that, similar to decision trees, use attributes to automatically identify subgroups, but in a different way, try to identify extreme segments by the difference of treatments, identifying segments that are particularly influential.

Once one tries to define profiles of behaviors that lead to abandonment, it is necessary to find characteristics or attributes that somehow allow to trace those profiles. Work related to retention in sports services ([1] [5] [11] [12] [13]) allows systematizing and identifying attributes necessary to characterize users and their behavior, both those who continue to use the services and those who leave, and that can be found in

the databases of the computer systems of these facilities: 1) demographic attributes such as age and gender; (2) Attributes related to contracting the service such as contracted frequency, number of months of enrollment and turnover (LTV); (3) Attributes related to frequency such as actual frequency, average frequency and number of days without visiting the premises; (4) Other attributes related to quality of service such as complaints or other manifestations of dissatisfaction, contacts made, assessments of the physical condition or any other type of evaluation.

3 Preparation of Data

Considering the need to obtain records with the attributes of the type indicated in the referred groups, it was considered a Lisbon sport facility database that uses a market application (e@sport) to which Extract, Transform and Load (ETL) processes were applied, as described by Trujillo [14], considering the entire history of users who were (or still are) enrolled in aquatic or fitness activities between 01/June/2014 and 31/October/2017.

According to the first step of Database Marketing is intended to create a data warehouse with a fact table where will reside the relevant attributes that will support the predictive model. Since the performance of some Machine Learning techniques is limited to the manipulation of values of a certain type or the performance itself is influenced by the range of values [15], in addition to the attributes directly mapped from the source database, some attributes have been transformed, discretized through numeric-symbolic conversions, or created new attributes that derive from classifications and transformations made on the original data or attributes. As so the ETL process resulted in the construction of a fact table in the data warehouse with 51 relevant attributes, although only forty-five have valid data. Relevant attributes, such as those related to the quality of the service were not filled due to lack of data. The attributes considered are presented in Table 1:

Table 1. Considered attribute groups.

Group	Attributes
(1) Demographic	Age (2 attributes), Gender, References (2 attributes), Distance to the facility
(2) Service level agreement	Number of months of enrollment (3 attributes), Turnover (2 attributes), Free Use, Attended activities (10 attributes), Number of activities attended, Contracted frequency (2 attributes), Number of renewals
(3) Frequency	Number of days without attendance (3 attributes), Average frequency (3 attributes), Total number of frequencies (2 attributes), Number of classes (2 attributes), Average frequency of classes (2 attributes), Ratio (real frequency/contracted frequency) (2 attributes), Training duration (2 attributes)
(4) Service quality	Number of contacts established, Indications of dissatisfaction (3 attributes), Number of manifestations of dissatisfaction, Last response NPS, Number of assessments of physical condition, Number of prescriptions

Table 1 shows that some attributes have variations that correspond to derivations that aim to discretize the value of the original attribute, and sometimes more than one method has been used. The Hughes method [16] (classification A - attributes whose name starts by *class*) was used in attributes *Number of months of enrollment*, *Turnover*, *Number of days without attendance*, *Average frequency, Total number of frequencies*, *Number of classes* and *Average frequency of classes*.

Through the indications obtained in the referenced literature, variant attributes were also added in a second classification (classification B - attributes whose name begins with *class2*) from the base attributes: *Days without frequency*, *Age*, *Number of months of enrollment*, *Average frequency*, *Ratio (real frequency/contracted frequency)* and *Training duration*.

In addition to the referred operations on the attributes, situations of missing values were also corrected through the strategy of removing the respective records.

Since most users of the sports facility practice aquatic or fitness activities, the users were grouped in three different fact tables according to the activities that they practiced during their frequency. After the execution of all ETL processes the final number of records is presented in Table 2.

Table 2. Number of users in data warehouse fact tables

Users	In Aquatic activities	In Fitness activities	Total number of users
Active	1226	803	1927
Dropouts	1697	4926	6454
Total	2923	5729	8381

4 The Predictive Model

To build and validate the predictive model we used Microsoft SQL Server Analysis Services Designer Ver. 13.0.1701.8. This product provides a classification algorithm, Microsoft Decision Trees, based on decision trees that are, according to several authors ([7] [10] [17]), adequate and most used in studies related to retention where we need to predict a class from a nominal attribute. In this case, the *Withdrawal* attribute is the attribute that we want to predict (the target attribute). By definition we considered the value 1 to classify a Dropout user and the value 0 for an Active user. In addition, decision trees produce human readable results which is very useful in this case.

The algorithm used evaluates the available attributes by punctuating each attribute according to the information it provides and proposes the construction of models based on the most scoring attributes. Since some attributes result from different forms of classification or discretization of the same characteristic, the proposed models eventually use redundant attributes. Gama *et al.* [15] states that since the process of constructing a tree selects the attributes to use, they result in models that tend to be quite robust in relation to the addition of irrelevant and redundant attributes. However, it is desired to obtain models with significant predictive capacity and that at the same time lead to actionable profiles redundant attributes should be avoided.

The elimination of the redundant attributes of the proposed models resulted in several models in which adjustments were made to obtain shallower trees with leaves that always have a number of examples greater than fifty. Table 3 present the evaluation metrics for each model obtained with the Holdout method considering 70% of data for training and 30% for testing of the models.

Table 3. Evaluation metrics of Predictive Models with Holdout Method

Model	#Nodes	Depth	Accuracy	Sensitivity	Specificity	Precision	F-Score
Ret71	30	6	87.90%	92.69%	72.53%	91.54%	92.11%
Ret81	37	6	87.90%	92.69%	72.53%	91.54%	92.11%
Ret91	45	7	87.58%	93.11%	69.85%	90.83%	91.95%
Fit71	18	5	87.94%	92.14%	63.64%	93.61%	92.87%
Fit81	24	5	88.40%	93.30%	60.08%	93.11%	93.21%
Fit91	22	5	87.94%	92.14%	63.64%	93.61%	92.87%
Aq71	12	4	87.91%	86.36%	90.03%	92.19%	89.18%
Aq81	20	5	88.14%	91.70%	83.29%	88.21%	89.92%
Aq91	18	5	88.14%	91.70%	83.29%	88.21%	89.92%

If it is not possible to consider a substantially better model among the models created, the choice on the model to be used falls on the model Ret71 created on the basis of all the users and that presents a tree in which the attributes used are less redundant and have fewer nodes (Principle of Parsimony / Occam's Razor) since it reduces complexity, minimizes the possibility of overfitting and facilitates the creation of actions in the next phase.

Fig. 1 presents the correlation diagram between the attributes used in the chosen model that results the tree shown in Fig. 2.

Fig. 1. Correlation diagram between the attributes used in Model Ret71

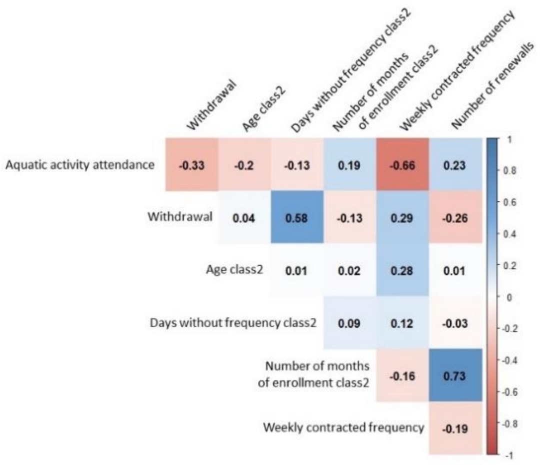

In each leaf of the tree there are examples that correspond to users quitting and examples that correspond to active users. The relationship between these quantities defines, on each leaf, a probability's threshold of withdrawal for the set of rules that define it. It is thus possible to draw dropout profiles from the rules on each leaf that shows a dropping rate above a considered threshold.

Fig. 2. Ret71 Model Decision Tree

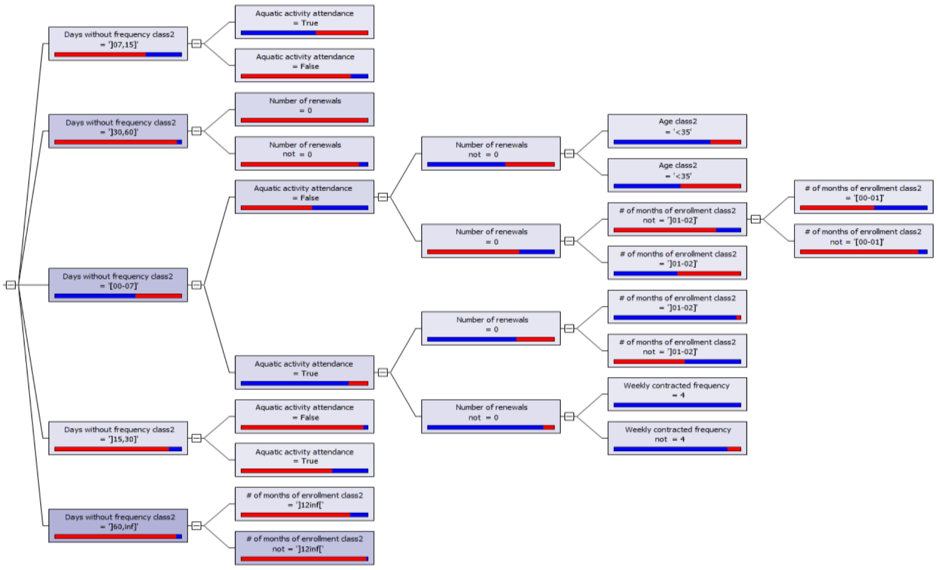

Considering, for example, a 90% dropout threshold, we found in the decision tree of Fig. 2 some leaves with upper thresholds, from which we can construct the profiles of Table 4.

5 Loyalty: planning the actions

In order to concentrate the efforts to increase the retention rate in groups more susceptible to abandonment of the sport, a bottom-up approach was adopted, according to Gorgoglione [18], and it is proposed the application of loyalty actions on the segmentation obtained with the predictive model referred to in the previous section, which obeys the segment utility criterion according to Kotler [19], which indicates that segmentation is only useful if the segments meet five criteria: they are measurable, substantial, accessible, differentiable and actionable.

For each of the actionable profiles obtained with the predictive model, a flow of conditionally sequential actions must be defined in which, at first, all the users that present this profile are the target of the first action. Secondly, only the users who did not change their behavior are the target of the second action, and finally, in a third moment, only the users who did not change their behavior after the first and second actions are the target of the third action.

Table 4. Dropout profiles obtained from the decision tree of the Ret71 model

Tree branch	Dropout profile
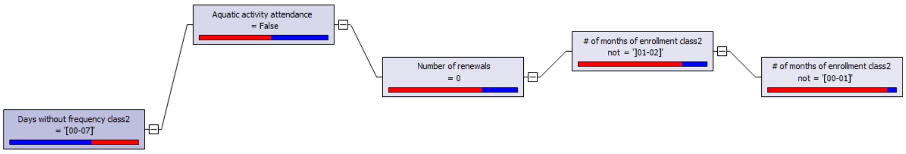	
Profile A – 92.66%	User that does not visit sport facilities for less than 7 days, does not attend water activities, never has renewed and their enrollment has more than two months old
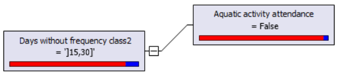	
Profile B – 96.22%	User who does not visit the facilities between 16 and 30 days and does not attend aquatic activities
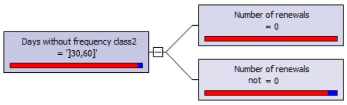	
Profile C – 96.38%	User who does not visit the facilities between 31 to 60 days
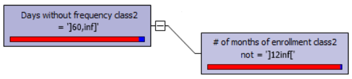	
Profile D – 98.42%	A user who has not visited the facilities for more than 60 days but whose enrollment is less than 12 months

If the change in characteristics and behavior of the user no longer places it on the leaf of the tree where it was initially placed, the user is no longer the target of this sequence or flow of actions. If this change fits into a target segment of another workflow, then the user is now framed in the flow of actions provided for the new profile.

Since it is expected that at each stage at least some of the target users of the corresponding action will change their behavior in order to not become dropouts, it is proposed to implement the workflow in three stages starting with the email, where the cost is practically zero, followed by the use of SMS and finally the personal contact, thus constructing a pyramid chaining, as shown in Fig. 3.

In addition to the cost, it is important to take into account the capacity of personalization and feedback and therefore many authors ([19] [20] [21]) attribute to email and phone great potential in promoting actions whose goal is to increase loyalty and retention rate.

Since the decision tree created by the algorithm may have multiple leafs where the withdrawal threshold is larger than indicated, multiple workflows with messages appropriate to the profile in which they affect can be designed and implemented.

The possible actions to be applied at each stage of the workflow can be grouped according to their purpose into four main groups: a) informative actions, with personalized information about the schedules that the user can attend and about the benefits included in the contracted service of which may not be making use; b) satisfaction

perception actions, namely Net Promoter Score (NPS) surveys, reasons for absences and other quality surveys; c) offer of benefits, namely at the level of free attendance of classes of other activities not included in the contracted service, substitution classes, participation in master classes or other events, free use of sports venues, gifts or vouchers; d) withdrawal of subscription and/or updating of consents under the General Data Protection Regulation (GDPR).

Fig. 3. Loyalty actions pyramid

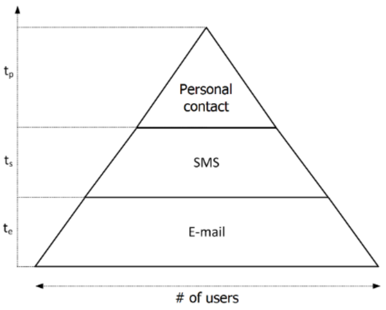

6 Loyalty evaluation: A/B tests

After defining the model described above, it is necessary to evaluate its effectiveness according to the following hypotheses:

H0: After performing the loyalty actions, the number of dropouts is the same as if no loyalty actions had been taken;

H1: After performing the loyalty actions, the number of dropouts is lower than if the loyalty actions had not been carried out;

The determination of which of the hypotheses occurs in a certain confidence interval will allow us to conclude whether or not there is a causal relationship between the application of the actions to the constructed profiles and the reduction of the number of withdrawals. It is proposed to validate hypotheses by planning experiments with the configuration indicated in Table 5.

Table 5. Experimental planning

Groups		Email		SMS		Personal	
R_t	O_{t1}	X_1	O_{t2}	X_2	O_{t3}	X_3	O_{t4}
R_c	O_{c1}		O_{c2}		O_{c3}		O_{c4}

The experiments are constructed through the implementation of A/B tests and evaluated through the chi-square method, which will allow to gauge a statistical conclusion for the problem in question.

A/B tests are used by manipulating a causal variable and where it is sought to determine the impact of this manipulation on two different groups of individuals, one experiencing the experiment and the other does not. In this case, the groups are creat-

ed by splitting the target users of the actions directed to a profile in two groups of users: those on whom the loyalty actions are applied, the test group (t), and another group on which they will not be applied, the control group (c).

For the creation of groups it is proposed that their constitution be made at the beginning of the experiment by dividing in equal parts the users who present the profile defined by the leaf of the tree on which the set of actions is intended to be applied. Since the users that present the profile have different dropout probability, it is proposed to sort them in descending order of that probability, alternating their placement in each of the test and control groups until exhaust the users. This process, although not purely random, creates homogeneous and equivalent groups in terms of probability of withdrawal, which allows avoiding problems with validation in terms of selection for group composition and generalization of experience results [22].

The application of the chi-square method to these groups should have two objectives so it must be done in two steps. In a first step, the application of the method must be done after each action, which will allow a performance evaluation of the action in concrete. In a second step, the application of the method must be done after all actions have been taken to evaluate the model as a whole.

The application of the method can be carried out by the construction of a matrix where the observed (O) and expected values (E) of the number of users who have withdrawn and do not give up at the beginning and after the application of each action or after all actions have been taken, in the case of overall assessment.

If the result obtained for the overall evaluation of the model has a confidence level higher than 95%, H0 can be rejected and it can be concluded with statistical relevance that after performing the loyalty actions, the number of dropouts is lower than if no loyalty actions had been taken.

7 Conclusions

With this work we try to present a valuable contribution to increase loyalty and retention rates in regular sports services through the development of a system that generates actionable knowledge based on real data.

Based in database marketing concepts this Actionable Knowledge Discovery System is based on three steps:

- Use real data from the ERP and CRM systems to extract records and attributes that characterize the behavior of the users that dropout;
- To obtain segments and profiles through the construction of predictive models that allow in a certain threshold of certainty to differentiate the users who dropout from the other users;
- To interact with users who are about to give up by implementing loyalty actions specifically directed to the characteristics of the profiles where each user fit; the value-added of loyalty actions is measured using A/B tests.

This actionable knowledge discovery system was developed in Cedis enterprise and is being implemented in two final customers before GDPR came into effect.

References

1. Avourdiadou, S., Theodorakis, N.: The development of loyalty among novice and experienced customers of sport and fitness centres. Sport Management Review (17), 419–431 (2014).
2. AGAP, Barómetro 2016, (2016).
3. Cavique, L.: Relatório da Unidade Curricular de Database Marketing, 2005-2006, Escola Superior Comunicação Social Instituto Politécnico de Lisboa
4. Peppers, D., Rogers, M.: Managing Customer Relationships, (30097), Wiley (2004)
5. Howat, G., Assaker, G.: Outcome Quality in participant sport and recreation service quality models: Empirical results from public aquatic centres in Australia, Sport Management Review (19), 520-535 (2016)
6. Pinheiro, P., Cavique, L.: Determinação de padrões de desistência em Ginásios, Revista de Ciências da Computação (10), 33-60 (2015)
7. Pinheiro, P., Cavique, L.: Modelos para incremento da retenção em serviços desportivos regulares: Análise preditiva e ações de fidelização. 13ª Conferência Ibérica de Sistemas e Tecnologias de Informação (CISTI), 1-6 (2018)
8. Mahajan, V., Misra, R., Mahajan, R.: Review of data mining techniques for churn Prediction in Telecom, Journal of information and organizational research (39) 183-197 (2015)
9. Cavique, L.: Micro-Segmentação de Clientes com Base de Dados de Consumo: Modelo RM-Similis, Revista Portuguesa e Brasileira de Gestão (2) 72-77 (2003)
10. Siegel, E.: Predictive Analytics The power to predict who will click, buy, lie or die, Wiley (2013)
11. Surujlal, J., Dhurup, M.: Establishing and maintaining customer relationships in commercial health and fitness centres in South Africa, International Journal of Trade, Economics and Finance, (3) 14-18 (2012)
12. Gonçalves, C.: Variáveis internas e externas ao indivíduo que influenciam o comportamento de retenção de Sócios no Fitness, Podium Sport, Leisure and Tourism Review, (1) 28-58 (2012)
13. Frota, M.: Gestão da Retenção, Manual de Gestão de Ginásios e Health Clubs – Excelência no sector do Health & Fitness, 103-148 (2011)
14. Trujillo, J., Luján-Mora, S.: A UML based approach for modeling ETL processes in data warehouses, Conceptual Modeling ER (2813) 307-320 (2003)
15. Gama, J., Carvalho, A., Faceli, K., Lorean, A., Oliveira, M.: Extração de Conhecimento de Dados (2017)
16. Hughes, A.: Strategic Database Marketing, McGraw-Hill, ISBN: 9780071773485
17. Tan, P., Steinbach, M., Kumar, V.: Introduction to Data Mining, Pearson (2016)
18. Gorgoglione, M.: Beyond customer churn: generating personalized actions to retain customers in a retail bank by a recommender system approach, Journal of Intelligent Learning Systems and Applications (3) 90-102 (2011)
19. Kotler, P., Keller, K.: Marketing Management (22-4) (2009)
20. Pousttchi, K., Wiedemann, D.: A contribution to theory building for mobile marketing: categorizing mobile marketing campaigns through case study research, Mobile Business (2925) (2006)
21. Merisavo, M., Raulas, M.: The impact of e-mail marketing on brand loyalty, Journal of Product & Brand Management, (13-7) 498-505 (2004)
22. Smith, S., Albaum, G.: An introduction to Marketing Research, (2010)
